# Complete genome sequence of *Peptostreptococcus anaerobius* SB204, isolated from a human colonic adenocarcinoma

**DOI:** 10.1128/mra.00804-25

**Published:** 2025-10-17

**Authors:** Rutika Gavate, Martha A. Zepeda-Rivera, Dakota S. Jones, Kaitlyn D. LaCourse, Susan Bullman, Christopher D. Johnston

**Affiliations:** 1 Genomic Medicine, MD Anderson Cancer Center, Houston, Texas, USA; 2 Vaccine and Infection Disease Division, Fred Hutchinson Cancer Center 561181 , Seattle, Washington, USA; 3 Human Biology Division, Fred Hutchinson Cancer Center 551089 , Seattle, Washington, USA; 4 Immunology, James P. Allison Institute, MD Anderson Cancer Center, Houston, Texas, USA; University of Maryland, School of Medicine, Baltimore, Maryland, USA

**Keywords:** *Peptostreptococcus anaerobius*, colorectal cancer, PacBio, SMRT-Seq

## Abstract

We report the complete genome sequence of *Peptostreptococcus anaerobius* SB204, a strain isolated from the resected tumor of a treatment-naive patient with colorectal cancer. The genome comprises a single chromosomal contig of 2.15 Mbp with an overall GC content of 36.1%.

## ANNOUNCEMENT

*Peptostreptococcus anaerobius* was originally classified under *Streptococcus* in 1905 but reclassified to *Peptostreptococcus* in 1936 due to its distinct anaerobic growth and metabolic characteristics (1). It is a gram-positive, anaerobic, non-spore-forming coccus commonly found in the human gastrointestinal and genitourinary tracts (2–4). Although typically regarded as a commensal organism, it is implicated in a range of infections, including pelvic inflammatory disease (5), dental abscesses (6), intra-abdominal infections (7), and bacteremia (8). Here, we document the isolation and complete genome sequence of *P. anaerobius* strain SB204 from colorectal adenocarcinoma tissue of a treatment-naive female patient.

**Fig 1:**
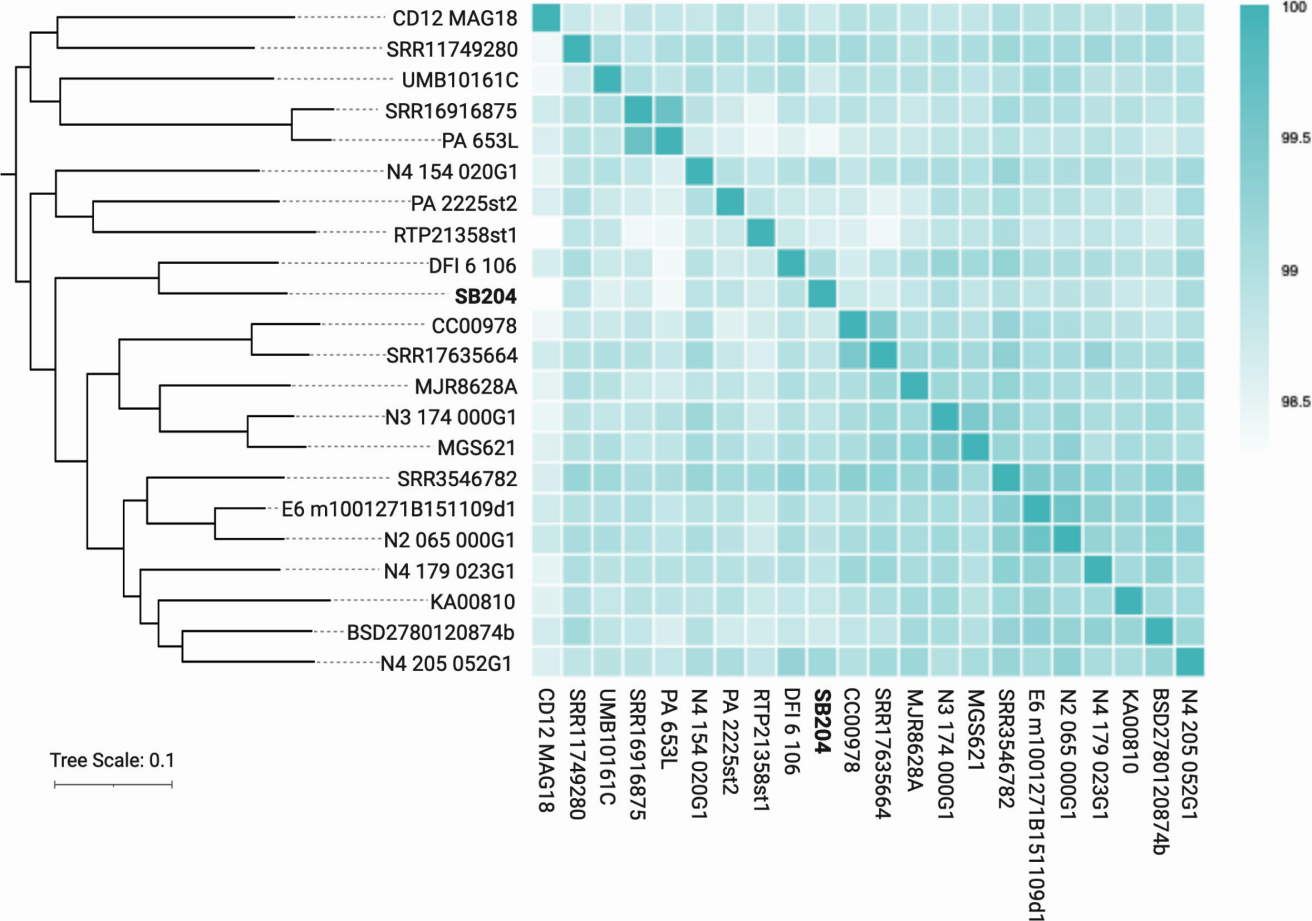
Heat map of ANI values among selected *P. anaerobius* genomes. (Left) Reference-free whole-genome phylogenetic tree of *P. anaerobius* genomes (*n* = 22) generated with kSNP4 (11) (kmer = 17, FCK = 0.658) with (right) corresponding heatmap of ANI values. Duplicate genomes (ANI > 99%) have been removed. ANI heatmap was generated with the pheatmap R package, RStudio version 2024.12.0+467. The tree was visualized using iTOL (12), and the figure was made with Biorender.

With written informed patient consent, approved by the Fred Hutchinson Cancer Center Institutional Review Board (protocol RG-1006552), SB204 was isolated from resected tumor specimen plated on Fastidious Anaerobic Agar (FAA; Oxoid, Thermo Fisher Scientific) supplemented with 10% defibrinated horse blood (Lampire Biological Laboratories, Fisher Scientific) and incubated under anaerobic conditions at 37°C for 48 h (AnaeroGen Gas Generating Systems, Oxoid, Thermo Fisher Scientific). BLAST analysis of the SB204 16S rRNA gene sequence determined initial taxonomic classification as *P. anaerobius*. High-molecular-weight genomic DNA was extracted using the MasterPure DNA Purification Kit (Epicentre, Lucigen). DNA concentration and quality were measured using the Qubit dsDNA BR Assay Kit (Thermo Fisher Scientific). DNA was sheared via G-tube (Covaris) as previously described (9) and size selected for fragments >5 kb using BluePippin (Sage Science). Sequencing library was prepared using the HiFi Assembly Kit (Pacific Biosciences). Single-molecule real-time (SMRT) sequencing (10) with base kinetics was performed on a PacBio Sequel I instrument with V3 sequencing chemistry (Pacific Biosciences). Sequencing reads were quality filtered and processed using microbial assembler in the Pacific Biosciences’ SMRTAnalysis pipeline (version 9.0.0.92188) with default parameters. Genome assembly was performed using Microbial Assembler, which included error correction and chromosomal rotation to place *dnaA* at the origin. Genome assembly showed 93,738 polymerase reads that were further partitioned into 591,799 mapped subreads, with an N50 value of 9,175 nucleotides and a total of 4,890,837,089 subread bases, yielding a mean coverage of 2,212×. Average nucleotide identity (ANI) analysis against publicly available *P. anaerobius* genomes (Table 1, Fig. 1) supported its species-level identification.

**TABLE 1:** Publicly available genome assemblies used for ANI analysis

Strain	Accession no.	Isolation source
CD12_MAG18	GCA_019013055.1	Mucosal tissue from Crohn’s disease patient
SRR11749280_bin.18_metaWRAP_v1.3_MAG	GCA_947086575.1	Vaginal metagenome
UMB10161C	GCA_030227555.1	Urine
SRR16916875_bin.10_metaWRAP_v1.3_MAG	GCF_946998135.1	Vaginal metagenome
PA 653-L	GCF_000178095.1	Biological product
N4_154_020G1_dasN4_154_020G1_abawaca.36	GCA_032475295.1	Infant feces
2225st2_D9_2225SCRN_200828	GCA_039756995.1	NA^*a*^
RTP21358st1_C2_RTP21358_211008	GCA_040909105.1	NA
DFI.6.106	GCA_024462755.1	Fecal sample
CC00978	GCA_964251445.1	NA
SRR17635664_bin.8_metaWRAP_v1.3_MAG	GCA_947253115.1	NA
MJR8628A	GCA_001563595.1	Vagina
N3_174_000G1_dasN3_174_000G1_concoct_28	GCA_032552295.1	Infant feces
MGS621	GCA_000431415.1	NA
SRR3546782_bin.52_CONCOCT_v1.1_MAG	GCA_937974095.1	NA
E6_m1001271B151109d1_201121	GCA_028326525.1	NA
N2_065_000G1_dasN2_065_000G1_abawaca.31	GCA_032557275.1	Infant feces
N4_179_023G1_dasN4_179_023G1_concoct_1	GCA_032472735.1	Infant feces
KA00810	GCA_001553145.1	Vagina
BSD2780120874b_170522_E5	GCA_015548685.1	NA
N4_205_052G1_dasN4_205_052G1_concoct_1	GCA_032471295.1	Infant feces

^*a*^ NA, not applicable.

Genome annotation using the NCBI Prokaryotic Genome Annotation Pipeline (13) (v6.1) identified 1,872 coding sequences, a GC content of 36.1%, and 78 RNAs. PADLOC (14) (v2.0.0) and CRISPR-Cas (15) analysis predicted the presence of multiple defense systems, including Type I-B CRISPR-Cas and Type I restriction-modification (RM) systems. To identify putative nucleotide motifs modified by the Type I RM system, we performed methylome analysis via REBASE (16), which identified m6A modification of TAAYN_5_CTC/CAGN_5_RRT. Analysis via the Comprehensive Antibiotic Resistance Database (17) (v4.0.1) identified antimicrobial resistance genes associated with putative resistance to fluoroquinolones (*sdrM*), glycopeptides (*vanT* and *vanW*), and tetracycline (*tetM*).

Currently, there are 51 publicly available *P. anaerobius* genome assemblies, although by ANI analysis, some appear to be duplicates. Notably, SB204 is the first complete genome sequence available for this species and the first reported strain from colorectal tumor tissue (Table 1). This isolate and its genome provide a novel resource for studying the clinical role(s) of this organism, particularly in the tumor microenvironment.

## Data Availability

SB204 raw sequencing reads were deposited in the NCBI Sequence Read Archive repository under accession number SRR33936918 as part of BioProject accession number PRJNA549513, and the RefSeq assembly accession number is GCF_050780165.1. The genome sequence was deposited in GenBank under the accession number CP096607.1. Base modification files were submitted with the GenBank accession, and methylome analysis is available at REBASE under organism number 93728.

## References

[1] Murdoch DA. 1998. Gram-positive anaerobic cocci. Clin Microbiol Rev 11:81–120. doi:10.1128/CMR.11.1.81 9457430 PMC121377

[2] Legaria MC, Nastro M, Camporro J, Heger F, Barberis C, Stecher D, Rodriguez CH, Vay CA. 2021. Peptostreptococcus anaerobius: pathogenicity, identification, and antimicrobial susceptibility. Review of monobacterial infections and addition of a case of urinary tract infection directly identified from a urine sample by MALDI-TOF MS. Anaerobe 72:102461. doi:10.1016/j.anaerobe.2021.102461 34626800

[3] Ng J, Ng LK, Chow AW, Dillon JA. 1994. Identification of five Peptostreptococcus species isolated predominantly from the female genital tract by using the rapid ID32A system. J Clin Microbiol 32:1302–1307. doi:10.1128/jcm.32.5.1302-1307.1994 8051260 PMC263676

[4] Riggio MP, Lennon A. 2002. Development of a PCR assay specific for Peptostreptococcus anaerobius. J Med Microbiol 51:1097–1101. doi:10.1099/0022-1317-51-12-1097 12466408

[5] Saini S, Gupta N, Aparna, Batra G, Arora DR. 2003. Role of anaerobes in acute pelvic inflammatory disease. Indian J Med Microbiol 21:189–192. doi:10.1016/S0255-0857(21)03071-1 17643017

[6] Siqueira JF Jr, Rôças IN. 2013. Microbiology and treatment of acute apical abscesses. Clin Microbiol Rev 26:255–273. doi:10.1128/CMR.00082-12 23554416 PMC3623375

[7] Alvarez JA, Baldonedo RF, Bear IG, Alvarez P, Jorge JL. 2004. Anaerobic liver abscesses as initial presentation of silent colonic cancer. HPB (Oxford) 6:41–42. doi:10.1080/13651820310015798 18333045 PMC2020643

[8] Minces LR, Shields RK, Sheridan K, Ho KS, Silveira FP. 2010. Peptostreptococcus infective endocarditis and bacteremia. Analysis of cases at a tertiary medical center and review of the literature. Anaerobe 16:327–330. doi:10.1016/j.anaerobe.2010.03.011 20371296

[9] Flores Ramos S, Brugger SD, Escapa IF, Skeete CA, Cotton SL, Eslami SM, Gao W, Bomar L, Tran TH, Jones DS, Minot S, Roberts RJ, Johnston CD, Lemon KP. 2021. Genomic stability and genetic defense systems in Dolosigranulum pigrum, a candidate beneficial bacterium from the human microbiome. mSystems 6:e0042521. doi:10.1128/mSystems.00425-21 34546072 PMC8547433

[10] Eid J, Fehr A, Gray J, Luong K, Lyle J, Otto G, Peluso P, Rank D, Baybayan P, Bettman B, et al.. 2009. Real-time DNA sequencing from single polymerase molecules. Science 323:133–138. doi:10.1126/science.1162986 19023044

[11] Hall BG, Nisbet J. 2023. Building phylogenetic trees from genome sequences with kSNP4. Mol Biol Evol 40:msad235. doi:10.1093/molbev/msad235 37948764 PMC10640685

[12] Letunic I, Bork P. 2021. Interactive Tree Of Life (iTOL) v5: an online tool for phylogenetic tree display and annotation. Nucleic Acids Res 49:W293–W296. doi:10.1093/nar/gkab301 33885785 PMC8265157

[13] Tatusova T, DiCuccio M, Badretdin A, Chetvernin V, Nawrocki EP, Zaslavsky L, Lomsadze A, Pruitt KD, Borodovsky M, Ostell J. 2016. NCBI prokaryotic genome annotation pipeline. Nucleic Acids Res 44:6614–6624. doi:10.1093/nar/gkw569 27342282 PMC5001611

[14] Payne LJ, Meaden S, Mestre MR, Palmer C, Toro N, Fineran PC, Jackson SA. 2022. PADLOC: a web server for the identification of antiviral defence systems in microbial genomes. Nucleic Acids Res 50:W541–W550. doi:10.1093/nar/gkac400 35639517 PMC9252829

[15] Couvin D, Bernheim A, Toffano-Nioche C, Touchon M, Michalik J, Néron B, Rocha EPC, Vergnaud G, Gautheret D, Pourcel C. 2018. CRISPRCasFinder, an update of CRISRFinder, includes a portable version, enhanced performance and integrates search for Cas proteins. Nucleic Acids Res 46:W246–W251. doi:10.1093/nar/gky425 29790974 PMC6030898

[16] Roberts RJ, Vincze T, Posfai J, Macelis D. 2015. REBASE--a database for DNA restriction and modification: enzymes, genes and genomes. Nucleic Acids Res 43:D298–D299. doi:10.1093/nar/gku1046 25378308 PMC4383893

[17] McArthur AG, Waglechner N, Nizam F, Yan A, Azad MA, Baylay AJ, Bhullar K, Canova MJ, De Pascale G, Ejim L, et al.. 2013. The comprehensive antibiotic resistance database. Antimicrob Agents Chemother 57:3348–3357. doi:10.1128/AAC.00419-13 23650175 PMC3697360

